# Internal traction using a preserved tissue bridge for defect closure during colorectal endoscopic full-thickness resection

**DOI:** 10.1055/a-2979-9265

**Published:** 2026-10-29

**Authors:** Futao Wu, Siqi Yang, Xiaoying Lu, Lifang Huang, Yuanning Ye, Kangkang Li, Aimin Li

**Affiliations:** 1 Guangdong Provincial Key Laboratory of Gastroenterology, Institute of Gastroenterology of Guangdong Province, Department of GastroenterologyNanfang Hospital, Southern Medical UniversityGuangzhouGuangdongChina; 2 Department of GastroenterologyNanfang Hospital (Zengcheng Branch), Southern Medical UniversityGuangzhouChina

A 55-year-old woman underwent colonoscopy for an incidentally detected colonic subepithelial lesion. Colonoscopy revealed an approximately 8-mm hemispherical lesion with smooth overlying mucosa at the hepatic flexure. Endoscopic ultrasonography demonstrated a 5.8×3.4-mm homogeneous hypoechoic lesion arising from the muscularis propria with an intact serosal layer, suggesting a gastrointestinal stromal tumor ( Fig. 1 ). Endoscopic full-thickness resection (EFTR) was therefore performed ( Video 1 ).

**Fig. 1:**
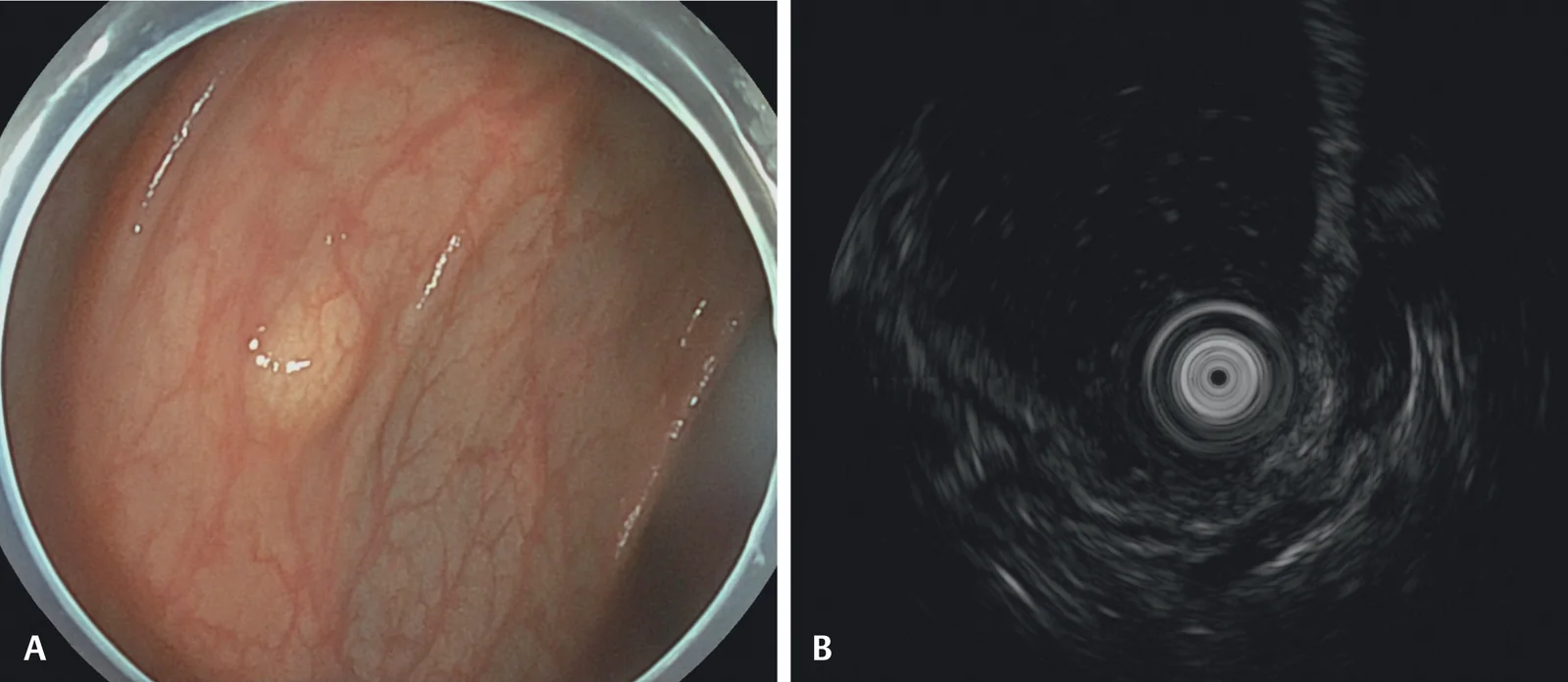
Endoscopic and endosonographic findings of the colonic subepithelial lesion. ( **a** ) White-light endoscopy showing a smooth subepithelial lesion at the hepatic flexure. ( **b** ) Endoscopic ultrasonography demonstrating a hypoechoic lesion arising from the muscularis propria.

**Video 1** Internal traction using a preserved tissue bridge for defect closure during colorectal endoscopic full-thickness resection. URI: .


After circumferential marking and submucosal injection, a central mucosal incision was made over the lesion to expose it while preserving the surrounding mucosa. Full-thickness dissection was then performed along one side of the lesion while intentionally preserving a narrow tissue bridge on the opposite side. Before complete detachment, the partially mobilized lesion was clipped to the opposite colonic wall to establish internal traction. Anchored by the clip and connected by the preserved tissue bridge, the lesion itself served as an internal traction anchor, progressively linearizing the full-thickness defect and facilitating sequential clip closure. After most of the defect had been closed, the preserved tissue bridge was divided with a snare, and the specimen was retrieved en bloc. The remaining defect was then completely closed with additional clips ( Fig. 2 ). The procedure was completed within 20 minutes without procedure-related adverse events. Histopathological examination demonstrated a spindle cell tumor ( Fig. 3 ).

**Fig. 2:**
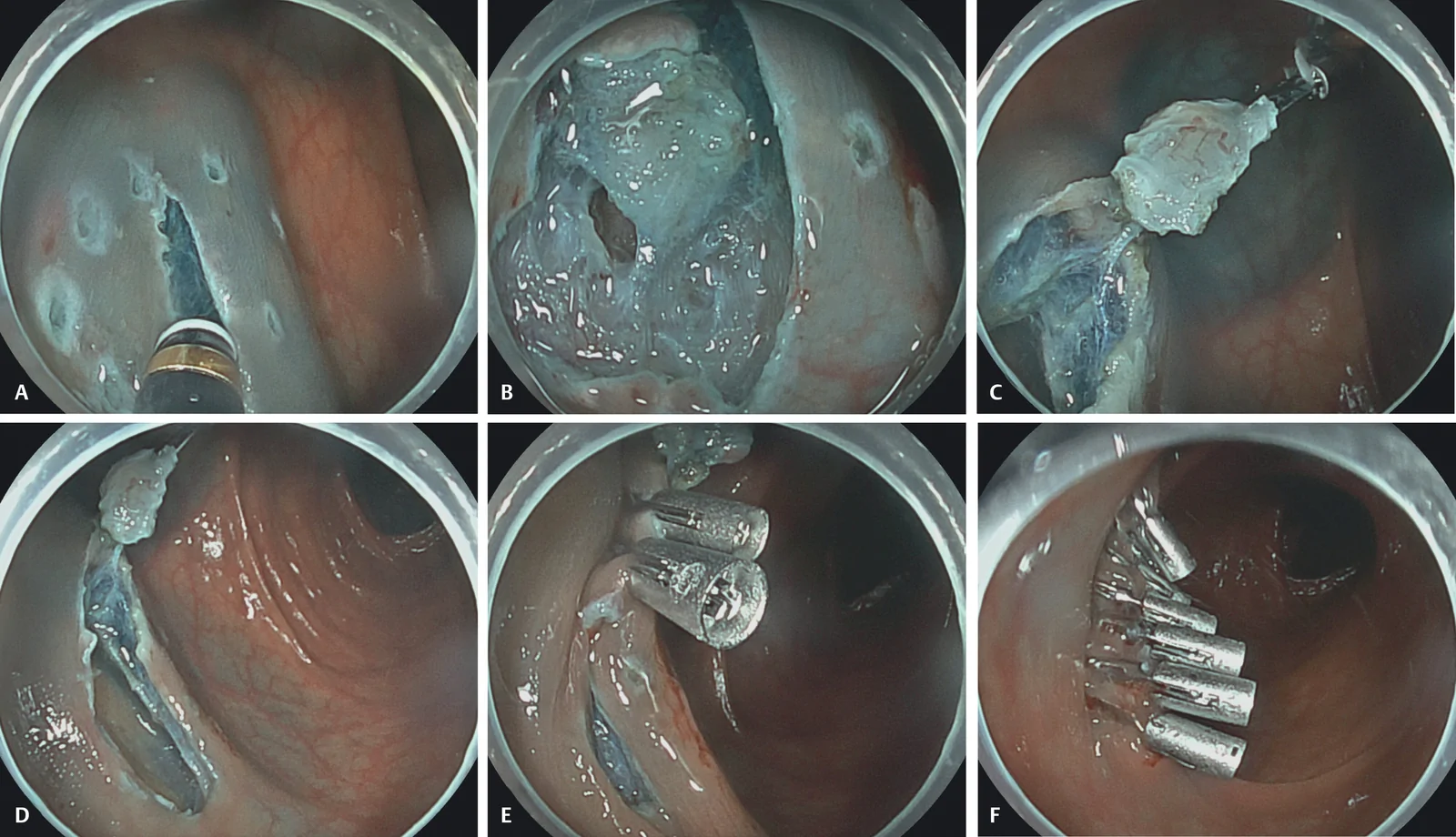
Internal traction using a preserved tissue bridge during colorectal endoscopic full-thickness resection. ( **a** ) Central mucosal incision to expose the lesion. ( **b** ) Full-thickness dissection with preservation of a tissue bridge. ( **c** ) Clip fixation of the partially detached lesion to the opposite colonic wall. ( **d** ) Linearization of the full-thickness defect by internal traction. ( **e** ) Sequential clip closure while maintaining internal traction. ( **f** ) Complete closure after division of the tissue bridge and retrieval of the lesion.

**Fig. 3:**
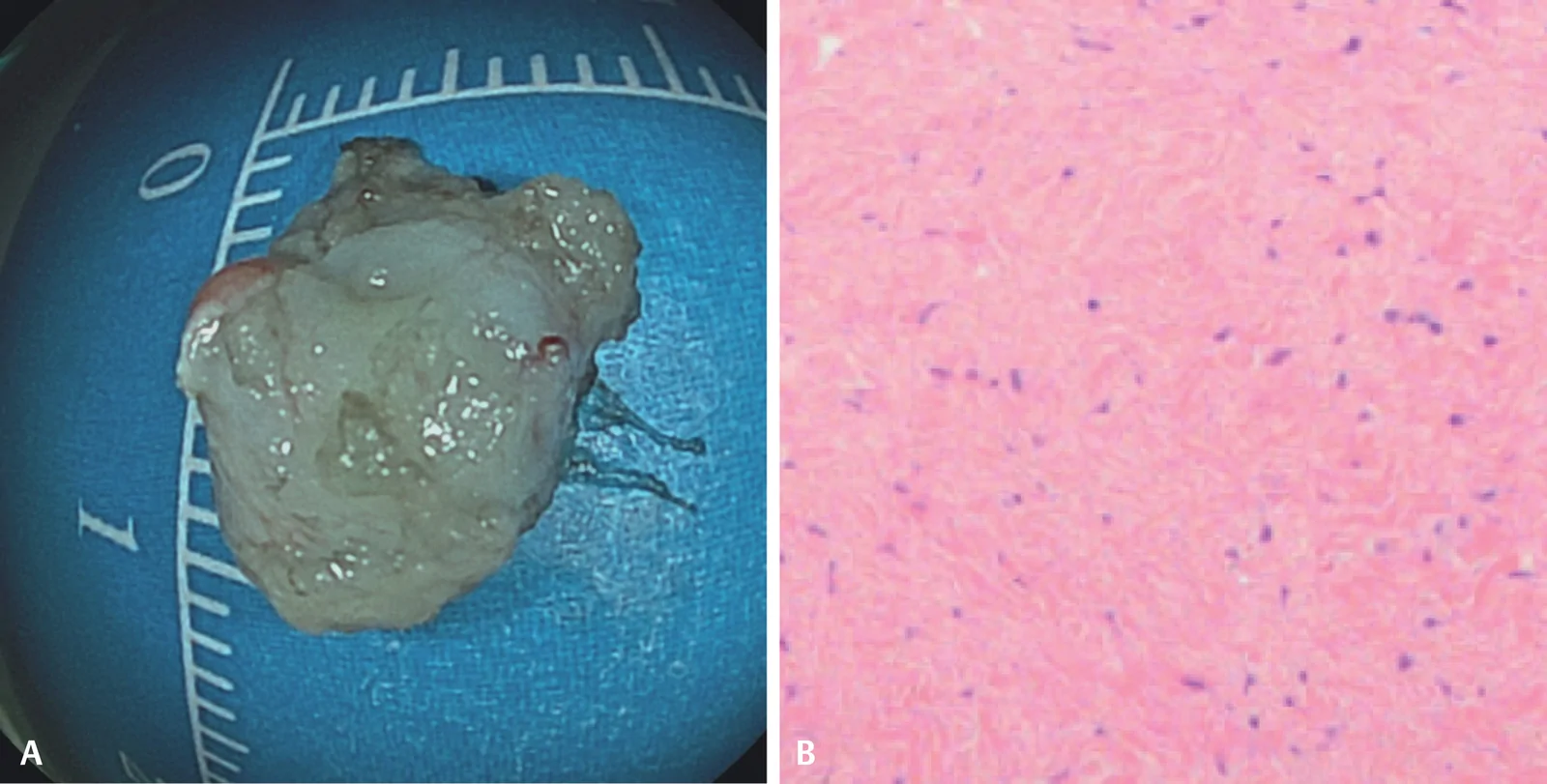
Resected specimen and histopathological findings. ( **a** ) En bloc resected specimen. ( **b** ) Histopathological examination showing a spindle cell tumor.

Closure after colorectal EFTR remains technically challenging. Several traction-assisted techniques have been reported to facilitate defect closure, although most require additional accessories or dedicated closure devices. 1​ ​2​ In contrast, our strategy uses the lesion itself as a temporary internal traction anchor by preserving a tissue bridge, thereby providing continuous traction without additional traction devices. This simple and practical approach facilitates defect linearization and clip approximation, potentially enabling efficient closure using readily available endoscopic accessories.

Endoscopy_UCTN_Code_TTT_1AQ_2AC

## Declaration of GenAI Use

The authors used ChatGPT (OpenAI) for language editing and to improve the clarity and readability of the manuscript. All contents were reviewed and approved by the authors, who take full responsibility for the final manuscript.

This study was supported by the President Foundation of Nanfang Hospital, Southern Medical University (2024B039); the Key Medical Discipline Program of Longgang District, Shenzhen, and the Guangzhou Clinical High-level, Major and Characteristic Medical Technology Project (2026P-ZD022); the Sanming Project of Medicine in Shenzhen (SZSM202105017); the National Natural Science Foundation of China (82373165); and the Guangdong Provincial Key Laboratory of Gastroenterology (2017B030314037).
